# Supplementary material for: Goal congruency dominates reward value in accounting for behavioral and neural correlates of value-based decision-making
Source: Nat Commun. 2019 Oct 29;10:4926. doi: 10.1038/s41467-019-12931-x (PMC6820735; doi:10.1038/s41467-019-12931-x)
Supplement: Supplementary file 1 — Supplementary Information [file 41467_2019_12931_MOESM1_ESM.pdf]

Supplementary Information
"Goal congruency dominates reward value in accounting for behavioral and neural correlates of value-based decision-making"

Frömer, Dean Wolf, and Shenhav

Supplementary Methods

Supplementary Study 1. 19 participants were recruited from Brown University and the general community, out of which one had to be excluded due to technical issues. Thus, the final sample includes 18 participants (61.1% female, M_{age} = 20.1, SD_{age} = 3.6). The procedures in Supplementary Study 1were identical to Study 1, except that participants only performed the Choose Worst condition (120 trials).

Supplementary Study 2. 15 participants were recruited from Brown University and the general community, out of which one had to be excluded due to technical issues. Thus, the final sample includes 14 participants (78.6 % female, M_{age} = 23.71, SD_{age} = 5.93). The procedures in Supplementary Study 2 were identical to Study 1, except that choices were incentivized. Following the individual item rating and immediately prior to the choice phase, participants were informed that they will receive one of the items they rated following the experiment and were asked to provide delivery information. They were informed that these options went into a lottery which they could bias with their subsequent choices. Depending on the choice task, they were then informed that choosing an option will increase (choose best) or decrease (choose worst) the probability of that option to be drawn in the final lottery.

Additional measures. The following measures were obtained in each experiment. With the exception of set appraisals, these additional evaluations are not of primary interest to the present study and were therefore not included in any analyses.

Affective Rating. Following the choices, participants sequentially viewed all choices again and for each set rated (1) how stressed/anxious that choice made them feel, (2) how confident they were in their choice, and (3) how much they liked the set as a whole (set appraisal).

Item reevaluation. At the end of the experiment, participants in Study S1and a subset of participants in Study 1 were asked to rate all items one more time with the same procedure as during subjective evaluation, but using a bipolar scale anchored at -10 ("dislike a great deal") and 10 ("like a great deal").

Individual difference measures. At the start of each session, participants completed the following personality inventories: the Behavioral Inhibition/Activation Scales (BIS/BAS), Neuroticism subscale of the NEO Five Factor Inventory, Intolerance for Uncertainty Scale, and Need for Cognition.

Supplementary Discussion

The slowing effect of overall value in the Choose-Worst condition is not dependent on a Choose-Best reference point or hypotheticality of choices. In Supplementary Study 1, participants only performed the Choose-Worst task. As in Studies 1-2, we found that RTs increased with increasing overall value (b = .52, t = 5.16, $p < .001$). As in these other studies, RTs were also faster for larger value differences (minimum value vs. average remaining, b = -.57, t = -11.10, $p < 2e-16$).

Supplementary Study 2's participants performed the same tasks as in Study 1 but, rather than engaging in hypothetical choices, these participants had the opportunity to receive one of

the items (products) from the study and their choices directly influenced which item they would receive. In spite of this difference, Supplementary Study 2's results were qualitatively identical to Study 1. RTs decreased with increasing overall value in choose best (b = -0.22, p = .002) but increased with increasing overall value in choose worst (b= 0.20, p = .005). Therefore, we once again observed a significant interaction between choice goal and overall value (b = -0.41, p < .001, Supplementary Table 1). Like in Study 1 and Study 2, goal values provided a parsimonious and sufficient account of the observed effects and there was no significant residual effect of overall reward value (b = -0.01, p = .838, Supplementary Table 2).

Supplementary Table 1

Fixed effects of Overall Reward Values vary by Choice Goal

	log RT				
Predictors	*Estimates*	*CI*	*t*	*df*	*p*
(Intercept)	1.70	1.52 – 1.88	18.77	15.00	**<0.001**
Value Difference	-0.61	-0.71 – -0.51	-12.05	1630.00	**<0.001**
Overall Value	-0.01	-0.11 – 0.09	-0.20	1635.00	0.838
Best - Worst Condition	0.08	-0.07 – 0.22	1.00	15.00	0.335
Overall Value: Best - Worst	-0.41	-0.61 – -0.22	-4.20	1489.00	**<0.001**
**Random Effects**					
σ^2	0.21				
$\tau_{00\ subj\_idx}$	0.10				
$\tau_{11\ subj\_idx.isChooseBestTrial2\text{-}1}$	0.07				
$\rho_{01\ subj\_idx}$	-0.30				
ICC	0.36				
N_{subj_idx}	14				
Observations	1644				
Marginal R^2 / Conditional R^2	0.066 / 0.404				

Note: Significant effects are highlighted in bold.

Supplementary Table 2

Model comparison for overall reward and overall goal value effects on RT

	R^2	*AIC*	*BIC*	*dAIC*	*Chi^2*	*p*
VD + $C_{b\text{-}w}$ (baseline)	0.33	2255	2293			
baseline + OV_{reward}	0.33	2257	2300	2	0.17	0.676
baseline + OV_{goal}	0.35	2239	2282	-18	17.77	**<0.001**
baseline + $C_{b\text{-}w}$: OV_{reward}	0.35	2241	2289	2	0.04	0.837

Note: For each study, models are compared sequentially, and dAIC is the difference in AIC of each model to the previous model. VD = Value Difference; OV = overall value; significant effects are highlighted in bold.

Supplementary Table 3

Fixed Effects Summary GLM-3: Reward and Goal Values within sub-regions of valuation network

	**BOLD Activity vmPFC**					**BOLD Activity vStr**				
Predictors	*Estimates*	*CI*	*t*	*df*	*p*	*Estimates*	*CI*	*t*	*df*	*p*
(Intercept)	-0.56	-0.70 – -0.42	-7.76	31.00	**<0.001**	-0.41	-0.55 – -0.27	-5.73	31.00	**<0.001**
Best - Worst Condition	-0.06	-0.21 – 0.09	-0.78	46.00	0.439	0.08	-0.10 – 0.27	0.90	37.00	0.374
Overall Reward Value	0.31	0.07 – 0.55	2.52	3747.00	**0.012**	0.28	0.08 – 0.48	2.80	4022.00	**0.005**
Relative Reward Value	0.05	-0.18 – 0.28	0.43	4202.00	0.669	-0.13	-0.32 – 0.07	-1.28	4237.00	0.200
Overall Goal Value	0.33	0.09 – 0.56	2.74	972.00	**0.006**	0.08	-0.12 – 0.28	0.76	2633.00	0.445
Relative Goal Value	0.33	0.09 – 0.57	2.70	4195.00	**0.007**	0.30	0.10 – 0.50	2.99	4133.00	**0.003**
RT	0.20	0.01 – 0.39	2.08	32.00	**0.045**	-0.17	-0.33 – -0.01	-2.12	32.00	**0.042**
**Random Effects**										
σ^2	3.20					2.15				
τ_{00}	0.13 $_{subj\_idx}$					0.13 $_{subj\_idx}$				
τ_{11}	0.05 $_{subj\_idx.isChooseBestTrial2-1}$					0.17 $_{subj\_idx.isChooseBestTrial2-1}$				
	0.11 $_{subj\_idx.cRT}$					0.08 $_{subj\_idx.cRT}$				
ρ_{01}	0.28					0.01				
	-0.09					0.09				
ICC	0.05					0.08				
N	30 $_{subj\_idx}$					30 $_{subj\_idx}$				
Observations	4270					4270				
Marginal R^2 / Conditional R^2	0.007 / 0.056					0.009 / 0.088				

Note: Significant effects are highlighted in bold.

Supplementary Table 4

PCC Activity tracks reward and goal congruency similarly to the valuation network

	BOLD Activity PCC				
Predictors	*Estimates*	*CI*	*t*	*df*	*p*
(Intercept)	-0.76	-0.90 – -0.63	-11.02	31.00	**<0.001**
Best - Worst Condition	0.04	-0.13 – 0.22	0.51	41.00	0.616
Overall Reward Value	0.25	0.03 – 0.48	2.21	3766.00	**0.027**
Relative Reward Value	-0.09	-0.30 – 0.13	-0.77	4216.00	0.443
Overall Goal Value	0.12	-0.11 – 0.34	1.03	1829.00	0.301
Relative Goal Value	0.43	0.21 – 0.66	3.77	3999.00	**<0.001**
RT	-0.16	-0.32 – -0.01	-2.08	31.00	**0.046**
**Random Effects**					
σ^2	2.81				
τ_{00} subj_idx	0.12				
τ_{11} subj_idx.isChooseBestTrial2-1	0.12				
τ_{11} subj_idx.cRT	0.04				
ρ_{01}	0.16				
	-0.01				
ICC	0.05				
N subj_idx	30				
Observations	4270				
Marginal R^2 / Conditional R^2	0.008 / 0.060				

Note: Significant effects are highlighted in bold.

Supplementary Table 5

Changes in value effects across the dorsal-ventral axis in striatum

	Estimates	*SE*	*t*	*df*	*p*
(Intercept)	-0.40	0.06	-6.26	29.61	**<0.001**
Region (linear)	-0.11	0.02	-6.28	25559.83	**<0.001**
Region (quadratic)	-0.08	0.02	-4.50	25559.83	**<0.001**
RT	-0.21	0.07	-3.00	30.15	**0.005**
Overall Reward Value	0.35	0.06	5.54	25479.49	**<0.001**
Relative Goal Value	0.20	0.07	2.99	25531.34	**0.003**
Region (linear): Overall Reward Value	-0.14	0.10	-1.41	25559.83	0.158
Region (quadratic): Overall Reward Value	-0.21	0.10	-2.10	25559.83	**0.036**
Region (linear): Relative Goal Value	0.24	0.11	2.19	25559.83	**0.029**
Region (quadratic): Relative Goal Value	0.02	0.11	0.20	25559.83	0.844

Note: Significant effects are highlighted in bold.

Supplementary Table 6

Testing for Differences in Reward and Goal Congruency related Activity between rACC and mOFC

	**Sum Sq**	**Mean Sq**	**NumDF**	**DenDF**	**F value**	**Pr(>F)**
Best - Worst Condition	1.6	1.6	1	226.88	1.67	0.197
Overall Reward Value	5.38	5.38	1	6676.4	5.63	**0.018**
Relative Reward Value	0.06	0.06	1	8475.2	0.07	0.796
Overall Goal Value	2.58	2.58	1	5966.15	2.7	0.101
Relative Goal Value	6.61	6.61	1	8405.74	6.91	**0.009**
Region	0.02	0.02	1	29.39	0.02	0.890
RT	0.23	0.23	1	28.54	0.24	0.628
Region: Best - Worst Condition	0.87	0.87	1	8440.16	0.91	0.341
Region: Overall Reward Value	0.21	0.21	1	2585.16	0.22	0.641
Region: Relative Reward Value	0.91	0.91	1	8455.28	0.95	0.330
Region: Overall Goal Value	0.05	0.05	1	8445.82	0.06	0.811
Region: Relative Goal Value	1.29	1.29	1	8358.15	1.34	0.246

Note: Significant effects are highlighted in bold.

Supplementary Table 7

Reward and Goal Congruency effects within rACC and mOFC

	BOLD Activity rACC					BOLD Activity mOFC				
Predictors	*Estimates*	*CI*	*t*	*df*	*p*	*Estimates*	*CI*	*t*	*df*	*p*
(Intercept)	-0.77	-0.87 – -0.67	-14.85	31.00	**<0.001**	-0.23	-0.34 – -0.12	-3.95	31.00	**<0.001**
Best - Worst Condition	-0.02	-0.13 – 0.09	-0.32	4087.00	0.751	-0.08	-0.20 – 0.03	-1.44	4154.00	0.150
Overall Reward Value	0.22	0.02 – 0.43	2.15	3253.00	**0.032**	0.12	-0.09 – 0.33	1.16	3562.00	0.247
Relative Reward Value	-0.06	-0.26 – 0.14	-0.61	4252.00	0.544	0.10	-0.11 – 0.31	0.95	4249.00	0.341
Overall Goal Value	0.02	-0.17 – 0.21	0.22	4216.00	0.830	0.22	0.03 – 0.42	2.24	4240.00	**0.025**
Relative Goal Value	0.18	-0.03 – 0.39	1.70	4203.00	0.089	0.24	0.03 – 0.45	2.19	4249.00	**0.028**
RT	-0.36	-0.53 – -0.18	-4.04	32.00	**<0.001**	0.30	0.10 – 0.49	2.98	33.00	**0.005**
**Random Effects**										
σ^2	2.37					2.50				
$\tau_{00\ subj\_idx}$	0.06					0.08				
$\tau_{11\ subj\_idx.cRT}$	0.11					0.17				
$\rho_{01\ subj\_idx}$	0.17					0.04				
ICC	0.03					0.04				
N_{subj_idx}	30					30				
Observations	4270					4270				
Marginal R^2 / Conditional R^2	0.014 / 0.047					0.009 / 0.052				

Note: Significant effects are highlighted in bold.

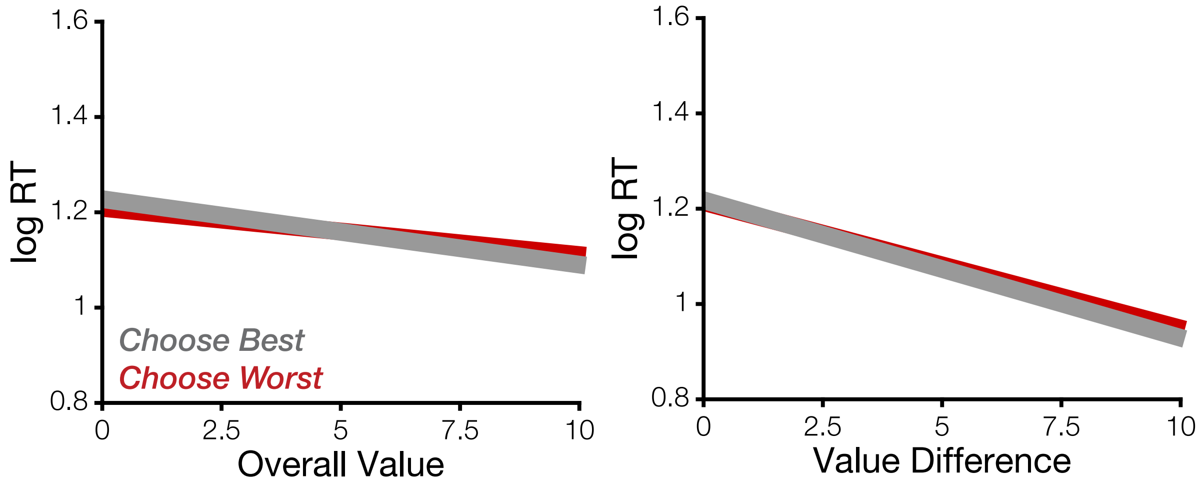

Supplementary Figure 1. LCA-simulated RTs based on reward values of the items. Increasing overall value (left) and value difference (right) lead to reduced RTs irrespective of choice goal. Thus the model cannot replicate the observed interaction of overall value and choice goal.

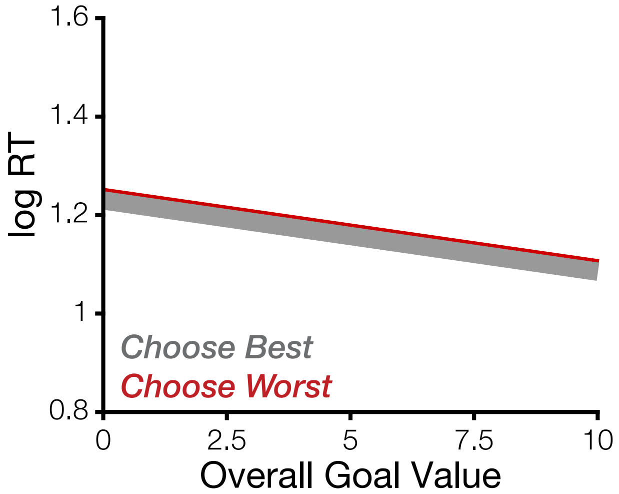

Supplementary Figure 2. LCA-simulated RTs based on goal values of the items. Increasing overall goal value captures RTs speeding across both choice goals.

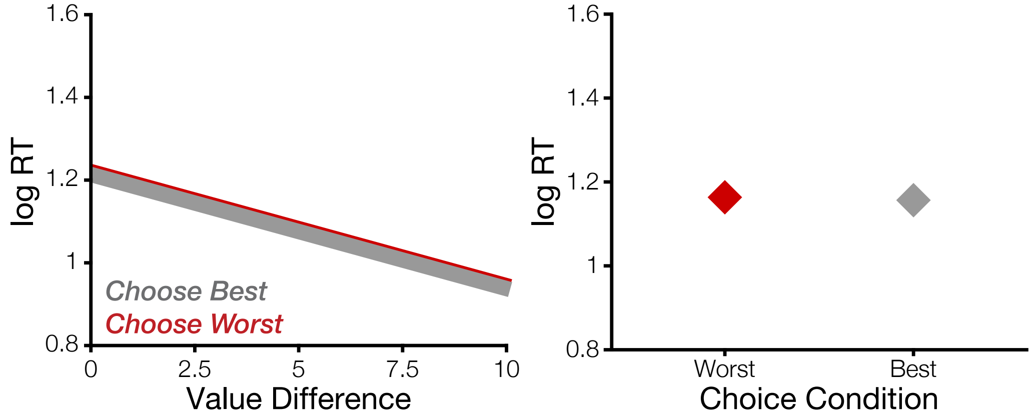

Supplementary Figure 3. LCA-simulated RTs based on goal values of the items replicates patterns in observed behavior. Increasing overall value (left) leads to reduced RTs for Choose Best, but increased RTs for Choose Worst. Increasing Value Difference leads to reduced RTs irrespective of choice goal.

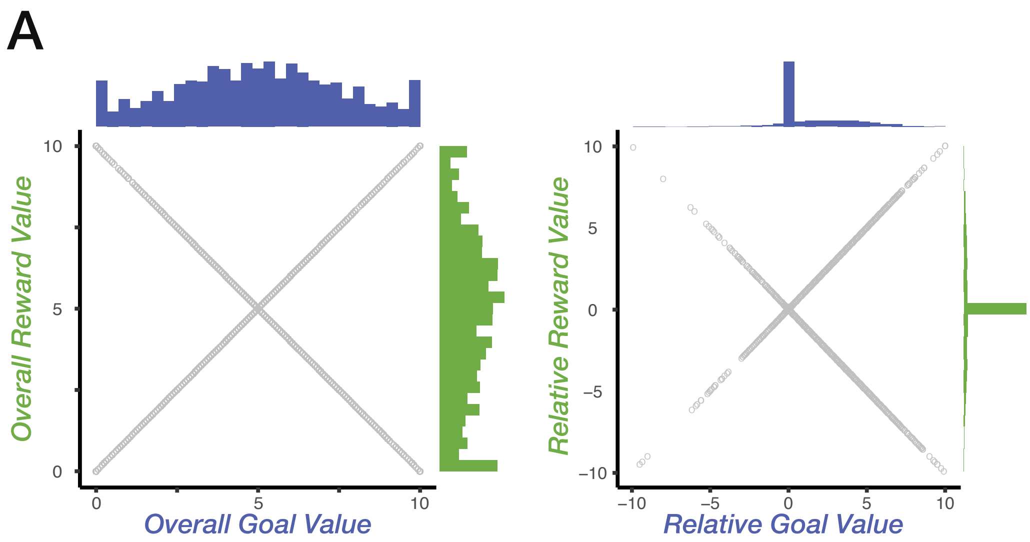

B

	Overall Goal Value	*Relative Reward Value*	*Relative Goal Value*
Overall Reward Value	**0**	**0.02**	**0.03**
Overall Goal Value		**–0.02**	**0.01**
Relative Reward Value			**0**

Supplementary Figure 4. Relationships between value-related predictors. **A.** Distributions of Overall and Relative Reward and Goal Values across all trials. **B.** Reward and Goal Values are orthogonal. Shown are Spearman correlation coefficients.

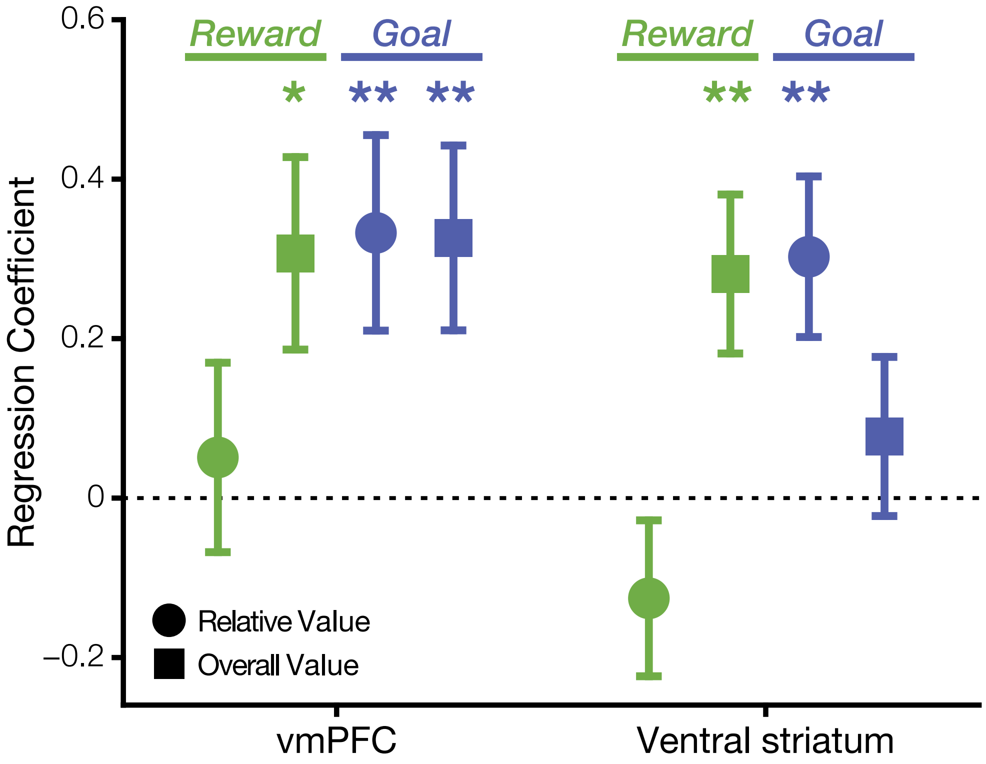

Supplementary Figure 5. Effects of Relative and Overall Reward and Goal Values within constituting regions of the valuation network.

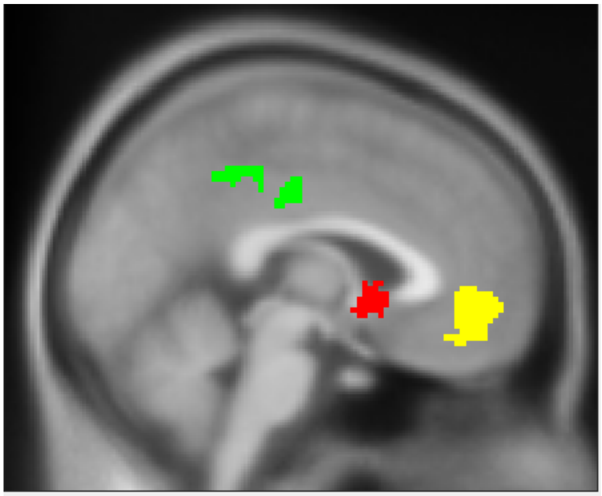

Supplementary Figure 6. Masks used for post-hoc analyses of vmPFC and vStr (yellow and red, respectively; drawn from Bartra et al., 2013) and PCC (green; drawn from Shenhav & Buckner, 2014).

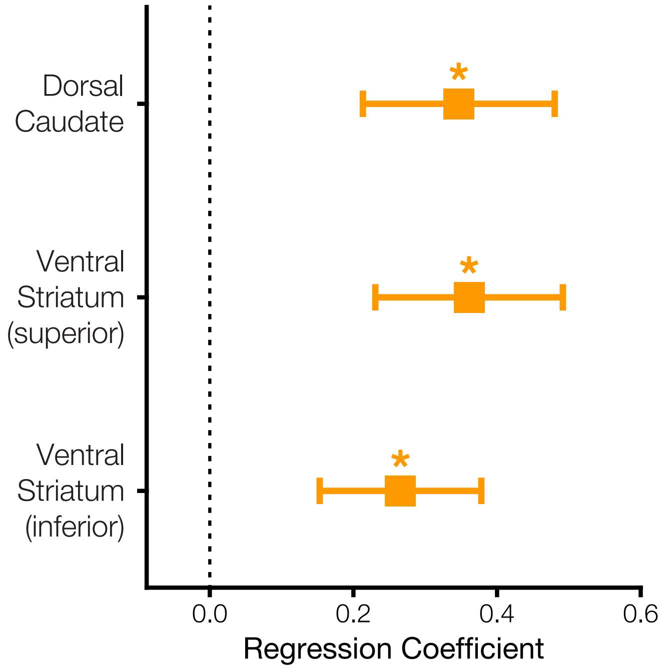

Supplementary Figure 7. Set appraisal (liking) effects along the striatum's dorsal to ventral axis.
