# Supplementary material for: Goal congruency dominates reward value in accounting for behavioral and neural correlates of value-based decision-making
Source: Nat Commun. 2019 Oct 29;10:4926. doi: 10.1038/s41467-019-12931-x (PMC6820735; doi:10.1038/s41467-019-12931-x)
Supplement: Supplementary file 3 — Reporting Summary [file 41467_2019_12931_MOESM3_ESM.pdf]

nature research

Corresponding author(s): Romy Froemer & Amitai Shenhav

Last updated by author(s): 2019 - 09 - 30

Reporting Summary

Nature Research wishes to improve the reproducibility of the work that we publish. This form provides structure for consistency and transparency in reporting. For further information on Nature Research policies, see Authors & Referees and the Editorial Policy Checklist.

Statistics

For all statistical analyses, confirm that the following items are present in the figure legend, table legend, main text, or Methods section.

n/a	Confirmed	
[ ]	[x]	The exact sample size (*n*) for each experimental group/condition, given as a discrete number and unit of measurement
[ ]	[x]	A statement on whether measurements were taken from distinct samples or whether the same sample was measured repeatedly
[ ]	[x]	The statistical test(s) used AND whether they are one- or two-sided<br>*Only common tests should be described solely by name; describe more complex techniques in the Methods section.*
[ ]	[x]	A description of all covariates tested
[ ]	[x]	A description of any assumptions or corrections, such as tests of normality and adjustment for multiple comparisons
[ ]	[x]	A full description of the statistical parameters including central tendency (e.g. means) or other basic estimates (e.g. regression coefficient) AND variation (e.g. standard deviation) or associated estimates of uncertainty (e.g. confidence intervals)
[x]	[ ]	For null hypothesis testing, the test statistic (e.g. *F*, *t*, *r*) with confidence intervals, effect sizes, degrees of freedom and *P* value noted<br>*Give P values as exact values whenever suitable.*
[x]	[ ]	For Bayesian analysis, information on the choice of priors and Markov chain Monte Carlo settings
[x]	[ ]	For hierarchical and complex designs, identification of the appropriate level for tests and full reporting of outcomes
[ ]	[x]	Estimates of effect sizes (e.g. Cohen's *d*, Pearson's *r*), indicating how they were calculated

Our web collection on statistics for biologists contains articles on many of the points above.

Software and code

Policy information about availability of computer code

Data collection	Data was collected using custom code in Psychtoolbox (Version 3.0.16) for Matlab (versions varied across testing sites; scanner: 2014a, lab: 2016b).
Data analysis	Data were analyzed using Matlab (2017b) and R(v. 3.6.1), SPM12.

For manuscripts utilizing custom algorithms or software that are central to the research but not yet described in published literature, software must be made available to editors/reviewers. We strongly encourage code deposition in a community repository (e.g. GitHub). See the Nature Research guidelines for submitting code & software for further information.

Data

Policy information about availability of data

All manuscripts must include a data availability statement. This statement should provide the following information, where applicable:
- Accession codes, unique identifiers, or web links for publicly available datasets
- A list of figures that have associated raw data
- A description of any restrictions on data availability

The datasets generated and analyzed during the current study are available from the corresponding author on reasonable request.

Field-specific reporting

Please select the one below that is the best fit for your research. If you are not sure, read the appropriate sections before making your selection.

[x] Life sciences [ ] Behavioural & social sciences [ ] Ecological, evolutionary & environmental sciences

For a reference copy of the document with all sections, see nature.com/documents/nr-reporting-summary-flat.pdf

Life sciences study design

All studies must disclose on these points even when the disclosure is negative.

Sample size	The sample sizes in Study 1 and 2 were 30 participants each.
Data exclusions	For Study 1, 37 participants were recruited. Of these, seven were excluded, one due to previous participation in a similar experiment, three due to incomplete sessions, and three due to insufficient variance in product evaluation, precluding the generation of sufficient choice sets. For Study 2, 31 participants were recruited and one was excluded due to an incomplete session.
Replication	The behavioral findings in Study 1 were replicated in Study 2, as well as Supplementary Study 2. The effects of reversed overall value effects under choose worst were replicated in Supplementary Study 1, testing this choice condition, only.
Randomization	All participants performed the same tasks. The order of choice blocks was counter-balanced based on the subject ID.
Blinding	There were no differential tests across participants, so blinding was not applicable.

Reporting for specific materials, systems and methods

We require information from authors about some types of materials, experimental systems and methods used in many studies. Here, indicate whether each material, system or method listed is relevant to your study. If you are not sure if a list item applies to your research, read the appropriate section before selecting a response.

Materials & experimental systems

n/a	Involved in the study	
[ ]	[ ]	Antibodies
[ ]	[ ]	Eukaryotic cell lines
[ ]	[ ]	Palaeontology
[ ]	[ ]	Animals and other organisms
[ ]	[x]	Human research participants
[ ]	[ ]	Clinical data

Methods

n/a	Involved in the study	
[ ]	[ ]	ChIP-seq
[ ]	[ ]	Flow cytometry
[ ]	[x]	MRI-based neuroimaging

Antibodies

Antibodies used	*Describe all antibodies used in the study; as applicable, provide supplier name, catalog number, clone name, and lot number.*
Validation	*Describe the validation of each primary antibody for the species and application, noting any validation statements on the manufacturer's website, relevant citations, antibody profiles in online databases, or data provided in the manuscript.*

Eukaryotic cell lines

Policy information about cell lines

Cell line source(s)	*State the source of each cell line used.*
Authentication	*Describe the authentication procedures for each cell line used OR declare that none of the cell lines used were authenticated.*
Mycoplasma contamination	*Confirm that all cell lines tested negative for mycoplasma contamination OR describe the results of the testing for mycoplasma contamination OR declare that the cell lines were not tested for mycoplasma contamination.*
Commonly misidentified lines<br>(See ICLAC register)	*Name any commonly misidentified cell lines used in the study and provide a rationale for their use.*

Palaeontology

Specimen provenance	*Provide provenance information for specimens and describe permits that were obtained for the work (including the name of the issuing authority, the date of issue, and any identifying information).*
Specimen deposition	*Indicate where the specimens have been deposited to permit free access by other researchers.*
Dating methods	*If new dates are provided, describe how they were obtained (e.g. collection, storage, sample pretreatment and measurement), where they were obtained (i.e. lab name), the calibration program and the protocol for quality assurance OR state that no new dates are provided.*

[ ] Tick this box to confirm that the raw and calibrated dates are available in the paper or in Supplementary Information.

Animals and other organisms

Policy information about studies involving animals; ARRIVE guidelines recommended for reporting animal research

Laboratory animals	*For laboratory animals, report species, strain, sex and age OR state that the study did not involve laboratory animals.*
Wild animals	*Provide details on animals observed in or captured in the field; report species, sex and age where possible. Describe how animals were caught and transported and what happened to captive animals after the study (if killed, explain why and describe method; if released, say where and when) OR state that the study did not involve wild animals.*
Field-collected samples	*For laboratory work with field-collected samples, describe all relevant parameters such as housing, maintenance, temperature, photoperiod and end-of-experiment protocol OR state that the study did not involve samples collected from the field.*
Ethics oversight	*Identify the organization(s) that approved or provided guidance on the study protocol, OR state that no ethical approval or guidance was required and explain why not.*

Note that full information on the approval of the study protocol must also be provided in the manuscript.

Human research participants

Policy information about studies involving human research participants

Population characteristics	Study 1: 76.7 % female, Mage = 20.3, SDage = 2.1<br>Study 2: 56.67% female, Mage = 21.87, SDage = 4.40<br>Supplementary Study 1: 61.1% female, Mage = 20.1, SDage = 3.6<br>Supplementary Study 2: 78.6 % female, Mage = 23.71, SDage = 5.93
Recruitment	Participants were recruited from Brown University and the general community via flyers and an online participant portal.
Ethics oversight	Brown University Institutional Review Board

Note that full information on the approval of the study protocol must also be provided in the manuscript.

Clinical data

Policy information about clinical studies

All manuscripts should comply with the ICMJE guidelines for publication of clinical research and a completed CONSORT checklist must be included with all submissions.

Clinical trial registration	*Provide the trial registration number from ClinicalTrials.gov or an equivalent agency.*
Study protocol	*Note where the full trial protocol can be accessed OR if not available, explain why.*
Data collection	*Describe the settings and locales of data collection, noting the time periods of recruitment and data collection.*
Outcomes	*Describe how you pre-defined primary and secondary outcome measures and how you assessed these measures.*

ChIP-seq

Data deposition

[ ] Confirm that both raw and final processed data have been deposited in a public database such as GEO.

[ ] Confirm that you have deposited or provided access to graph files (e.g. BED files) for the called peaks.

Data access links<br>*May remain private before publication.*	*For "Initial submission" or "Revised version" documents, provide reviewer access links. For your "Final submission" document, provide a link to the deposited data.*
Files in database submission	*Provide a list of all files available in the database submission.*
Genome browser session<br>(e.g. UCSC)	*Provide a link to an anonymized genome browser session for "Initial submission" and "Revised version" documents only, to enable peer review. Write "no longer applicable" for "Final submission" documents.*

Methodology

Replicates	*Describe the experimental replicates, specifying number, type and replicate agreement.*
Sequencing depth	*Describe the sequencing depth for each experiment, providing the total number of reads, uniquely mapped reads, length of reads and whether they were paired- or single-end.*

Antibodies	*Describe the antibodies used for the ChIP-seq experiments; as applicable, provide supplier name, catalog number, clone name, and lot number.*
Peak calling parameters	*Specify the command line program and parameters used for read mapping and peak calling, including the ChIP, control and index files used.*
Data quality	*Describe the methods used to ensure data quality in full detail, including how many peaks are at FDR 5% and above 5-fold enrichment.*
Software	*Describe the software used to collect and analyze the ChIP-seq data. For custom code that has been deposited into a community repository, provide accession details.*

Flow Cytometry

Plots

Confirm that:

[ ] The axis labels state the marker and fluorochrome used (e.g. CD4-FITC).

[ ] The axis scales are clearly visible. Include numbers along axes only for bottom left plot of group (a 'group' is an analysis of identical markers).

[ ] All plots are contour plots with outliers or pseudocolor plots.

[ ] A numerical value for number of cells or percentage (with statistics) is provided.

Methodology

Sample preparation	*Describe the sample preparation, detailing the biological source of the cells and any tissue processing steps used.*
Instrument	*Identify the instrument used for data collection, specifying make and model number.*
Software	*Describe the software used to collect and analyze the flow cytometry data. For custom code that has been deposited into a community repository, provide accession details.*
Cell population abundance	*Describe the abundance of the relevant cell populations within post-sort fractions, providing details on the purity of the samples and how it was determined.*
Gating strategy	*Describe the gating strategy used for all relevant experiments, specifying the preliminary FSC/SSC gates of the starting cell population, indicating where boundaries between "positive" and "negative" staining cell populations are defined.*

[ ] Tick this box to confirm that a figure exemplifying the gating strategy is provided in the Supplementary Information.

Magnetic resonance imaging

Experimental design

Design type	event-related design
Design specifications	2 blocks (one choose best and one choose worst) of 72 trials each, trial duration varied as a function of RT, ITI varied across trials (2-7s, uniformly distributed)
Behavioral performance measures	behavioral measures were choices and RT, in addition outside the scanner, value and subjective experience ratings were collected.

Acquisition

Imaging type(s)	functional
Field strength	3T
Sequence & imaging parameters	64-channel phase-arrayed head coil, using the following gradient-echo planar imaging (EPI) sequence parameters: repetition time (TR) = 2500 ms; echo time (TE) = 30 ms; flip angle (FA) = 90°; 3 mm voxels; no gap between slices; field of view (FOV): 192 x 192; interleaved acquisition; 39 slices. To reduce signal dropout in regions of interest, we used a rotated slice prescription (30° relative to AC/PC)
Area of acquisition	the whole brain was scanned.
Diffusion MRI	[ ] Used [x] Not used

Preprocessing

Preprocessing software	We used SPM12 to conduct realignement within participants, resampling to 2mm isotropic voxels, non-linear transformation to align with a canonical T2 template and spatial smoothing with a 6 mm full-width at half-max (FWHM) Gaussian kernel
Normalization	non-linear transformation to align with a canonical T2 template
Normalization template	We normalized to SPM12's T2 MNI template
Noise and artifact removal	We used a reweighted least squares approach (RobustWLS Toolbox) in our GLMs to minimize the influence of outlier time-points (e.g. due to motion).
Volume censoring	none

Statistical modeling & inference

Model type and settings	Preprocessed data were submitted to linear mixed-effects analyses using a two-step procedure. In the first step, we computed first-level general linear models (GLM) in SPM to generate BOLD signal change estimates for each trial and participant. GLMs modeled stick functions at the onset of each trial. Trials were concatenated across the two task blocks and additional regressors were included to model within-block means and linear trends. GLMs were estimated using a reweighted least squares approach (RobustWLS Toolbox) to minimize the influence of outlier time-points (e.g. due to motion). The obtained estimates were transformed with the hyperbolic arcsine function (to achieve normality), and then analyzed using LMMs using lme4 in R.<br>We complemented the ROI analyses with whole-brain GLMs. For these analyses, we computed first-level GLMs, modeling stick function at stimulus onsets, and parametric regressors for 1) choice goal, 2) reward-related OV, 3) goal-related OV, 4) reward-related chosen versus unchosen value and 5) goal-related chosen versus unchosen value. Regressors were de-orthogonalized to let them compete for variance. As above, trials were concatenated across the two task blocks, additional regressors were included to model within-block means and linear trends, and GLMs were estimated using RobustWLS. Second level random effects analyses on first-level estimates were performed using SPM with voxel-wise thresholds of p < .001 and cluster-corrected thresholds of p < .05.
Effect(s) tested	We tested the effects of overall and relative reward and goal value (continuous regressors) while controlling for choice type (best vs worst) and RT.

Specify type of analysis: ☐ Whole brain ☒ ROI-based ☐ Both

Anatomical location(s)	*Describe how anatomical locations were determined (e.g. specify whether automated labeling algorithms or probabilistic atlases were used).*
Statistic type for inference (See Eklund et al. 2016)	cluster wise
Correction	permutation

Models & analysis

n/a	Involved in the study
☒	☐ Functional and/or effective connectivity
☒	☐ Graph analysis
☒	☐ Multivariate modeling or predictive analysis
